# Cannabidiol exerts multitarget immunomodulatory effects on PBMCs from individuals with psoriasis vulgaris

**DOI:** 10.3389/fimmu.2024.1373435

**Published:** 2024-03-27

**Authors:** Cristina Pagano, Elena Ciaglia, Laura Coppola, Valentina Lopardo, Annunziata Raimondo, Monfrecola Giuseppe, Serena Lembo, Chiara Laezza, Maurizio Bifulco

**Affiliations:** ^1^ Department of Molecular Medicine and Medical Biotechnology, University of Naples Federico II, Naples, Italy; ^2^ Department of Medicine, Surgery and Dentistry “Scuola Medica Salernitana”, University of Salerno, Baronissi, Italy; ^3^ Section of Dermatology - Department of Clinical Medicine and Surgery, University of Naples Federico II, Naples, Italy; ^4^ Institute of Endocrinology and Experimental Oncology (IEOS), National Research Council (CNR), Naples, Italy

**Keywords:** psoriasis, CBD, natural killer, monocyte, dendritic cells, macrophage

## Abstract

**Introduction:**

The involvement of endocannabinoid system (ECS) in the inflammatory cascade, and the ability of phytocannabinoids, endocannabinoids and their synthetic analogues to modulate it has become an interesting research area for new therapeutic approaches in inflammatory skin diseases. Cannabidiol (CBD) appears to be the most promising among phytocannabinoids, due to the lack of psychotropic effects and low toxicity profile. Its anti-inflammatory action has been highlighted in different preclinical models, ranging from experimental colitis to arthritis and neuroinflammation. Our aim was to evaluate CBD immune-modulatory effects in peripheral blood mononuclear cells (PBMC) of psoriasis individuals with particular attention to both innate and adaptative immune arms.

**Methods:**

We performed *in vitro* immune functional experiments to analyze CBD action on various immune cells active in psoriatic lesions.

**Results:**

The results showed that CBD produced a shift from Th1 to Th2 response, while boosting cytotoxic activity of Natural Killer (NK) cells. Furthermore, it also exerted a potent action on monocyte differentiation as, after CBD treatment, monocytes from psoriatic individuals were unable to migrate in response to inflammatory stimuli and to fully differentiate into mature dendritic cells. Finally, a M2 skewing of monocyte-derived macrophages by CBD also contributed to the fine tuning of the magnitude of immune responses.

**Conclusions:**

These data uncover new potential immunomodulatory properties of this cannabinoid suggesting a possible therapeutic action in the treatment of multiple inflammatory skin diseases.

## 1 Introduction

Psoriasis is a chronic inflammatory disorder of the skin affecting approximately 1–3% of the population (1). It is characterized by hyperproliferation of the keratinocytes in the epidermis in regions such as elbow, scalp, and knee with inflammatory infiltrates, epidermal hyperplasia, dysregulated differentiation, and desquamation of keratinocytes (2). The inflammatory infiltrate consists mainly of dendritic cells, macrophages, and T cells in the dermis and neutrophils, with some T cells in the epidermis. Furthermore, psoriatic plaques are dominated by the presence of Th1 and Th17 lymphocytes, natural killer (NK) cells, and neutrophils with few B cells. The causes of psoriasis have not yet been fully clarified, but there are data which suggests that in psoriasis, the immune system is activated in an unbalanced way. The immunopathogenesis of psoriasis is complex and not fully understood due to its complex inflammatory cascade, triggered by innate immune cells (keratinocytes, dendritic cells, NKT cells, macrophages) and at the same time, it evolves and is enhanced by their interaction with adaptive immune cells (T lymphocytes). Indeed, it is described that the proinflammatory cytokine cascade is triggered by plasmacytoid dendritic cells (pDC) activated by complexes of DNA and the antimicrobial peptide cathelicidin (LL-37), released by injured keratinocytes. Activated dendritic cells produce numerous cytokines, including IL-12 and IL-23, which in turn promote the expansion of some particular types of T lymphocytes called Th1 and Th17. Th17, fundamental in the immunopathogenesis of psoriasis, produce cytokines that stimulate the proliferation of keratinocytes, such as IL-17, or of a pro-inflammatory nature, such as TNFα. The sequence of these steps results in the hyperproliferation of keratinocytes, which leads to the formation of well-demarcated plaques of psoriasis and the creation of a pro-inflammatory environment. At the end of this process, keratinocytes produce substances that act on dendritic cells, thus closing the circuit and causing perpetual self-sustaining inflammation. The inflammatory cascade promotes angiogenesis contributing to the migration of immune cells into the lesion. The cytokines involved activate various intracellular pathways, which act on the transcription of key messenger genes. For example, cytokines such as IFN-γ, IL-12, IL-22, and IL-23 activate the JAK-STAT (from Janus Kinases—Signal Transducer and Activator of Transcription proteins) pathway and TNFα acts in part activating NF-κB, transcription factor important for cellular proliferation, differentiation and apoptosis. The T-lymphocyte and potential keratinocyte response also is modulated by the intracellular messenger cyclic adenosine monophosphate (cAMP). It has been observed that in the pathogenesis of psoriasis cAMP is hydrolyzed by the enzyme phosphodiesterase 4 (PDE-4) into 5′-antimicrobial peptide (AMP), reducing inhibition of the NF-κB signaling pathway by cAMP (3). The endocannabinoid system (ECS) consists of endocannabinoids, the enzymes responsible for their degradation, transporters of endocannabinoids and cannabinoid receptors CB1 and CB2. Recent studies have described that endocannabinoid system is found in keratinocytes and other cellular components such as fibroblasts, sebaceous glands, hair follicles, and melanocytes (4). It is involved in maintaining skin homeostasis, barrier formation and regeneration, and its dysregulation can contribute to several skin disorders, as atopic dermatitis, psoriasis, acne, and various cancers (5). Several studies have described that the endocannabinoid anandamide (AEA) is able to reduce the levels of various chemokines (TNFα and IL-23) in keratinocyte cell lines, and of IL-17 in co-cultures of lymphocytes and keratinocytes. These effects were prevented by a CB1 receptor antagonist (6). Moreover, CB2, CB1 and PPARγ receptors appear to be the most involved with a pivotal role in psoriasis. The validity of phytocannabinoids has been demonstrated in various pre-clinical studies improving symptoms in psoriatic individuals (7). Phytocannabinoids (PCBs) are the cannabinoids obtained from the Cannabis sativa L. plant (C. sativa). One of the main phytocannabinoids is the cannabidiol (CBD), a non-psychotropic compound which has a low binding affinity for CB1 and CB2 receptors, but through the modulation of multiple targets, it regulates several biological activities, as antioxidant, neuroprotective effects and various inflammatory signaling pathways (8). Recent studies have demonstrated that CBD inhibits keratinocyte proliferation and modulates antioxidant and anti-inflammatory activity in these cells. CBD can decrease the oxidative capacity of cells, inhibiting the generation of ROS. Furthermore, it causes an increase in the level of antioxidant proteins, including superoxide dismutase (SOD), and prevents oxidative modifications of lipids and proteins that may be responsible for the development of psoriasis (6). In this ex-vivo study we have examined the CBD immune-modulatory effects in peripheral blood mononuclear cells (PBMC) of psoriatic individuals compared to untreated PBMC. Briefly, CBD was able to significantly counteract inflammatory signals from LPS and PHA stimuli while boosting cytotoxic activity of Natural Killer (NK) cells. These stimulations are used to induce distinct types of immune responses, namely innate response (with LPS), and lymphocyte proliferation (with PHA). Furthermore, CBD also exerted a potent action on monocyte differentiation as, after CBD treatment, monocytes from psoriatic individuals were unable to migrate in response to inflammatory stimuli and to fully differentiate into dendritic cells responsible for tuning the magnitude of immune responses. In conclusion, the therapeutic potential of CBD for a possible topical treatment of skin disease, such as psoriasis, is very promising.

## 2 Materials and methods

### 2.1 Drugs

Cannabidiol (CBD) was purchased from Sigma-Aldrich (Sigma-Aldrich; Cat# 90899), dissolved in DMSO (1mM) (Sigma-Aldrich, Cat#D8418) and added to cell culture at 10μM at different treatment times depending on the analysis conducted. Lipopolysaccharide (LPS) was purchased from Sigma-Aldrich (Sigma-Aldrich; Cat# L2630) and used at 1 µg/ml for 24 h in presence or/and absence of CBD (10μM). Phytohemagglutinin-M (PHA-M) was purchased from Sigma-Aldrich (Sigma-Aldrich; Cat# 11082132001) and used at 5 µg/ml.

### 2.2 Donor cohorts

In this study, a group of n=8 individuals were recruited: n= 4 healthy controls and n = 4 psoriatic individuals. Peripheral blood (n = 4) from psoriatic individuals and (n= 4) healthy control were collected at the Outpatients Clinic of the Dermatology Department of the University of Salerno. Individuals mean age was 25+/-10, out of which there were 3 males and 1 female, controls were age and sex matched. Individuals were in at least 1 month wash out from any systematic treatment at the time of sample collection. Peripheral blood mononuclear cells (PBMCs) were extracted from whole blood by Ficoll density gradient (Histopaque®-1077, Sigma-Aldrich). After separation, PBMC were collected and washed for the subsequent experiments (monocytes, natural killer cells, dendritic cells and macrophages isolation). Experiments were conducted in 96-well plates. PBMCs or PBMCs-derived cells (NKs, monocytes) were seeded at 2*10^6^ cells/mL in RPMI-1640 (Gibco®, ThermoFisher Scientific) supplemented with 10% (v/v) fetal serum bovine (FBS, Gibco®, ThermoFisher Scientific), 1% (v/v) penicillin-streptomycin (Aurogene), 1% (v/v) MEM non-essential amino acids (MEM NEAA, Gibco®, ThermoFisher Scientific), 1% (v/v) sodium pyruvate (Aurogene).

All participants signed an informed consent for the management of personal anamnestic data and blood samples. The study was approved by the local ethical committee (prot num. 0037908 year 2021) and conducted in accordance with the ethical principles deriving from the Declaration of Helsinki.

### 2.3 Natural killer cells isolation

Peripheral Blood Mononuclear Cells (PBMCs) were extracted from whole blood by density gradient (Ficoll). After separation, PBMC were collected and washed for the subsequent experiments. Natural killer cells were isolated from psoriatic individuals’ and healthy donors’ PBMCs by immunomagnetic procedure (human NK Cell Isolation Kit, #130-092-657, Miltenyi Biotec) following the manufacturer’s protocol. NKs were treated with CBD (10μM) or with vehicle alone (DMSO 0,1%) in presence or absence of IL-2 stimulus (200IU/ml) and cell suspensions were used for subsequent flow cytometry analysis.

### 2.4 *In vitro* monocyte migration assay

Following PBMCs separation from whole blood using the methodology described above, CD14+ cells were separated using specific anti-CD14 micro-beads (CD14 MicroBeads human, Miltenyi Biotec). Isolated CD14+ monocytes were treated with CBD (10μM) or with vehicle alone (DMSO 0,1%) for 24 hours. For chemotaxis assay, 8 μm pore size transwell filters were used. Cell suspensions were placed in the transwell insert, and chemoattractants (10% FBS/RPMI-1640 supplemented with Monocyte Chemoattractant Protein-1 (MCP-1, 0,05 μg/mL) added in the well, incubated at 37°C for 18h to allow migration of the cells through the filter into the well. Cells were harvested and cell number determined using flow cytometry. Cells that had passed through the filter were harvested, washed, and incubated with 10 μL of fluorescein isothiocyanate (FITC)-labelled anti-CD14 antibody.

### 2.5 Generation of monocyte-derived dendritic cells and Th1/Th2 polarization assay

Following PBMCs separation from whole blood using the methodology described above, CD14+ cells were separated using specific anti-CD14 micro-beads (CD14 MicroBeads human, Miltenyi Biotec). Monocyte-derived dendritic cells were obtained by culturing CD14+ cells in 10% FBS RPMI 1640, 50 ng/ml GM-CSF and 1000 U/ml IL-4. After 6-8 days of culture, monocyte-derived DCs were terminally differentiated by incubation with LPS at 1 µg/ml (Sigma-Aldrich) for 24 h in presence of CBD (10μM) or with vehicle alone (DMSO 0,1%). Monocytes-derived DCs were harvested, to perform subsequent analysis.

### 2.6 Macrophage generation

CD14+ monocytes were positively selected from healthy donors’ and psoriatic individuals’ PBMCs by an immunomagnetic procedure (Miltenyi Biotec). Then, CD14+ cells were induced to differentiate in M1 or M2 macrophages using reagents included in the CellXVivo™ Human M1 or M2 Macrophage Differentiation Kit (R&D system), in presence of CBD (10μM) or with vehicle alone (DMSO 0,1%).

### 2.7 CD107a degranulation assay

Activated NK cells were cultured in complete medium at 37°C, 5% CO_2_ with or without K562 cells at 1:1 E:T ratio in the presence of PE-conjugated CD107a/IgG1 antibody in U-bottom 96well plates. After 1 h, Brefeldin A (5 μg/ml; Sigma-Aldrich) was added to cultures for an additional 3 h of incubation. At this time, cells were collected, washed with PBS with 2% FBS, stained with anti-CD56 PE-Cy5 (BD Biosciences) and anti-CD3 FITC (BD Biosciences), and analyzed by flow cytometry.

### 2.8 Cytokine detection

PBMCs from psoriatic individuals were treated with CBD (10μM) or with vehicle alone (DMSO 0,1%). 500 μL of cell lysate was run on each array of the Proteome Profiler Human XL Cytokine Array Kit (#ARY022B, R&D Systems). Data shown are from a 15-minute exposure to X-ray film. Profiles of mean spot pixel density were created using a transmission-mode scanner and the image analysis software ImageJ. PBMCs from psoriatic individuals were treated with CBD (0-10μM) or with vehicle alone (DMSO 0,1%), in presence or absence of PHA. PBMCs-conditioned media TNFα and IFNγ levels were determined using Human TNFα ELISA Kit (#E-EL-H0109, Elabscience) and Human IFN-γ ELISA Kit (#E-EL-H0108, Elabscience), respectively, following the manufacturer’s protocol.

### 2.9 Cytofluorimetric analysis

Natural killer cells from psoriatic individuals and healthy donors were stained with mAb against human CD57-FITC, CD69-PE, CD56-Cyc, CD3-APC, NKG2D, KIR3DL1, CD107a, CXCR3, CCR5.

Monocytes from psoriatic individuals were stained with mAb against human CD14, CD16, CD68, CD69, CCR7, CXCR3, CD62L, CX3CR1, CCR5, CCR2. Mature dendritic cells from psoriatic individuals were stained with mAb against human CD86, CD1a, CD3, IFNγ and IL-4.

Macrophages from psoriatic individuals and healthy donors were stained with mAb against human CD86, CD14, CD163, CCR5.

After 30 min incubation at 4°C in the dark, cells were washed, centrifuged, and resuspended in staining buffer for the flow cytometry analysis. For each test, cells were analyzed using a FACSVerse flow cytometer (BD Biosciences).

### 2.10 BrdU assay

For proliferation analysis, incorporated BrdU was detected by the BrdU cell proliferation assay (Roche, Basel, Switzerland) according to the manufacturer’s instructions. HaCaT cells were seeded in a flat bottom 96-well plates at a density of 5×10^3^ cells/well in 100μl of culture medium for 24 h. After treatment with CBD (1.5µM - 40µM) and DMSO as vehicle (0,1%), BrdU was added (100 µM), and then fixative/denaturing solution was added for 30 min. Anti-BrdU-POD was added for 90’, after being diluted according to instructions. Cells were then washed, substrate solution (100 μl/well) was added. Absorbance was measured using a Synergy HT Microplate Reader (BioTek Instruments Inc.) at the wavelength of 450nm.

### 2.11 Statistical analysis

Statistical analysis was performed in all the experiments shown by using the GraphPad prism 9.0 software for Windows (GraphPad Software). For each type of assay, obtained data from multiple experiments are calculated as mean ± SD and analyzed for statistical significance using, for independent groups, or one ANOVA and two ANOVA followed by Bonferroni correction for multiple comparisons. p-values *p < 0.05, **p < 0.01, ***p < 0.001, ****p< 0,0001 versus control, represented by cells treated with vehicle (DMSO 0,1%) were considered significant. All experiments were performed in triplicate and repeated three times.

## 3 Results

### 3.1 CBD impacts on the cytotoxic and migratory activity of PBMCs from psoriatic individuals

**Figure 1:**
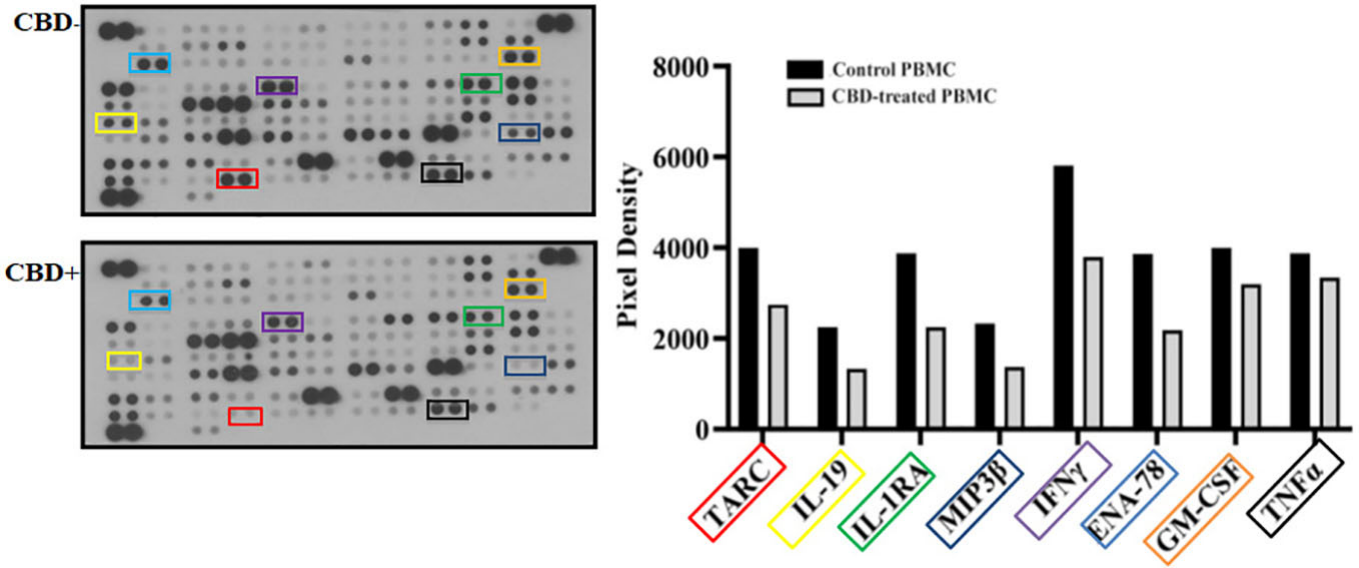
Cytokine profile analysis of PBMCs from psoriatic individuals. PBMCs not treated (CBD-, DMSO 0,1%) or treated with CBD+ (10μM) for 18 hours. The intensity of the positive control (reference spot) was considered 100% and the protein expression was expressed as a percentage of the intensity of the reference spots on the array. Spots related to TARC, MIP, IL-24, IL-19, IL-1RA, IFNγ, GM-CSF, ENA-78 are bordered in the representative array blots on the left and their expression is indicated in the histogram graph on the right.

**Figure 2:**
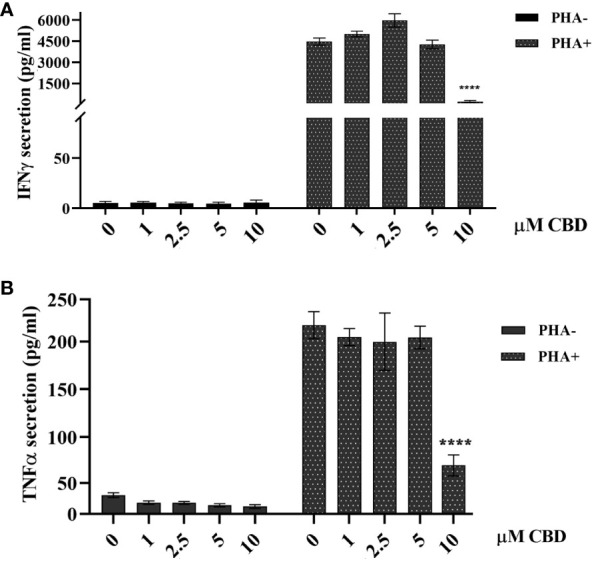
Effect of CBD on PBMCs’ release of TNFα and IFNγ from psoriatic individuals. Histograms report the levels of IFNγ **(A)** and TNFα **(B)** secretion quantified by ELISA assay. Psoriatic individuals’ PBMCs were treated with CBD (0-10μM) or with vehicle alone (DMSO 0,1%) for 18h hours in presence or absence of the activation stimulus PHA. Multiple comparisons statistically significant are indicated (ANOVA; ****p< 0,0001).

**Figure 3:**
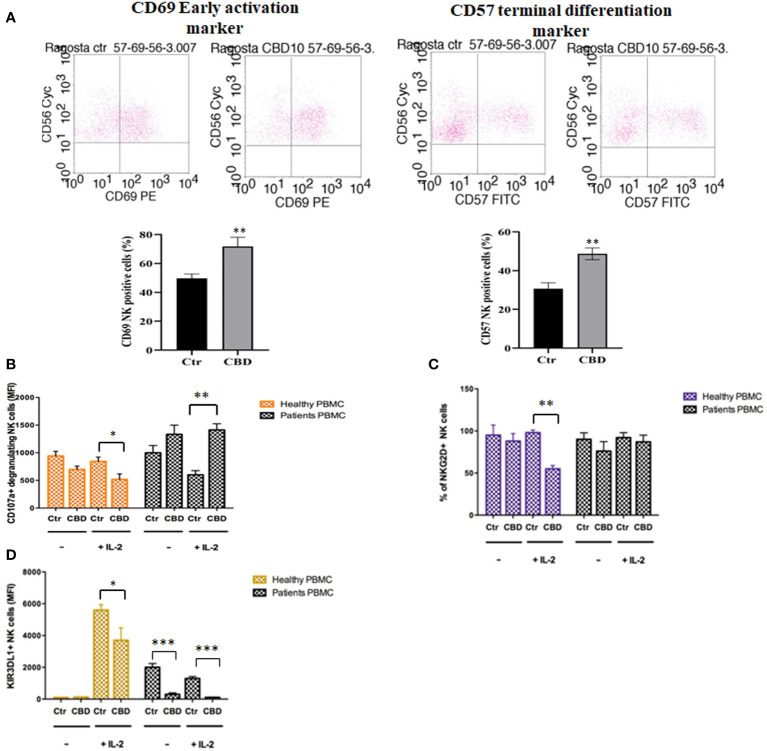
CBD affects the degranulating and inhibitory activity of Natural killer cells. Natural killer cells were isolated as indicated in Materials and methods section. Healthy donors’ and psoriatic individuals’ NKs (2*10^6^/ml in complete RPMI) were treated with CBD (10μM) or with vehicle alone (DMSO 0,1%) for 18 hours. **(A)** The panel shows a representative flow cytometry dot plot reporting the CD69 (early activation marker of NKs) expression on gated CD56 positive cells. Below, bar graph shows the flow cytometry histogram profile of CD69 protein levels at the cell surface of CD56+ NKs. **(B-D)** The flow cytometry histograms show the mean fluorescence intensity (MFI) of CD107a (marker of NKs degranulation), NKG2D (NK activator cell receptor) and KIR3DL1 (NK inhibitory cell receptor), respectively, in CD56 positive NK cells, after exposing NKs to CBD (10μM) for 18h hours in presence or absence of IL-2 (200IU/ml). We gated on low side scatter lymphocytes and then used CD3 and CD56 to identify NK cells (CD56+ CD3−). (ANOVA; *p< 0,05; **p< 0,01; ***p< 0,001).

**Figure 4:**
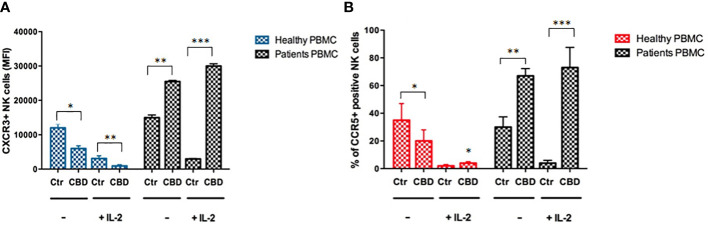
CBD impacts the expression of chemokine receptors on NK cells. The flow cytometry bar charts show respectively the percentage of **(A)** CXCR3 positive cells and **(B)** CCR5 positive cells on gated CD56 positive NK cells, after exposing healthy donors’ and psoriatic individuals’ NK cells, stimulated for 18h hours in presence or absence of IL-2 (200IU/ml) at a concentration of 2*10^6^/ml in complete RPMI medium. (ANOVA; *p< 0,05; **p<0,01; ***p< 0,001).

As immune-mediated disease, the complex pathogenesis of psoriasis involves the crosstalk among immune cells mainly through cytokines and mediators that are currently the target of existing biologic drugs (9). Considering little is known about the role of CBD in psoriasis-related immunological dysfunction, we started with assessing the effect of CBD on the cytokine release of control and CBD-treated PBMCs from psoriatic individuals. Before proceeding with the experiments, we evaluated the toxicity of CBD. For this purpose, we used a line of immortalized human keratinocytes (HaCAt cells) and performed a BrdU incorporation assay. As can be seen from **Supplementary Figure 1**, CBD is well tolerated by keratinocytes but at concentrations higher than 15 μM, it inhibits DNA synthesis compared to cells treated with vehicle alone (DMSO 0,1%), showing an IC_50 = _20 μM. Therefore, we used a concentration of 10 μM CBD also used by other investigators to study the immunomodulatory effect of CBD (10). The results of the proteome profile array highlighted that CBD at 10 μM leads to the reduction of TARC, MIP, IL-19, IL-1RA, IFNγ, GM-CSF, ENA-78, TNFα in psoriatic individuals’ PBMCs, compared to PBMCs treated with vehicle alone (DMSO 0,1%) (**Figure 1A**). The reduced inflammatory burden of CBD-treated psoriatic individuals’ PBMCs led us to specifically investigate the secretion levels of two inflammatory cytokine IFNγ and TNFα using a CBD response curve (0-10 μM). In response to phytohemagglutinin (PHA) stimulus, we found that only CBD at concentration of 10 μM significantly decreased the release of IFNγ and TNFα from PBMCs of psoriatic individuals (IFNγ: Control: 4400 pg/mL ± 1,5, CBD (10 µM) 215 pg/mL ± 0,2; TNFα: Control: 245 pg/mL± 0,7, CBD (10 µM) 60 pg/mL ± 1,2) (**Figure 2**). Among PBMCs, the production of IFNγ and TNFα is mainly functionally linked to the cytolytic and immunoregulatory activity of Natural killer cells. Thus, we hypothesized a putative involvement of NKs cells as cell target of the CBD effect described above. To this end, NKs were isolated from psoriatic individuals’ and/or healthy donors’ total PBMCs for comparison. To solely characterize the immunophenotype of NK cells in psoriatic individuals’, under basal conditions, by applying the FACS gating on CD3-CD56+ NK cells, we firstly found the increase of the early marker of cell activation CD69 on NK cells from psoriatic individuals treated with CBD (10 μM) compared to NK cells treated with vehicle alone (DMSO 0,1%) (CD69: Control: 43%± 1,2, CBD (10µM) 72% ± 3,5), and an increase of CD57 terminal differentiation marker (CD57: Control 37% ± 1,5, CBD (10µM) 45% ± 2) (**Figure 3A**). Furthermore, isolated NKs from psoriatic individuals’ and healthy donors were treated with CBD or with vehicle alone (DMSO 0,1%), in presence or absence of IL-2, as canonical NK activating stimulus, and then analyzed by flow cytometry. Overall, we found a) CBD potentiates the degranulating activity of NK cells from psoriatic individuals, but not in healthy donors, by evaluating the CD107a mobilization assay (**Figure 3B**); b) CBD did not affect the percentage of activated NKG2D positive NK cells of psoriatic individuals (**Figure 3C**); c) CBD significantly downregulates the expression of the inhibitory receptor KI3DL1 both in healthy and psoriatic individuals (**Figure 3D**). Meanwhile, CBD treatment can upregulate chemokine receptors on NK cells from psoriatic individuals, in detail CXCR3 and CCR5 receptors known to be involved in the recruitment and NK cell homing (**Figures 4A, B**).

### 3.2 The migratory ability of psoriatic individuals’ monocytes is affected by CBD treatment

**Figure 5:**
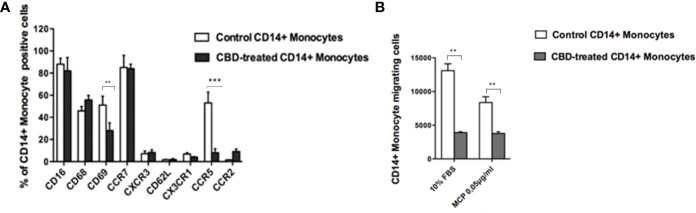
CBD alters monocytes’ migration to MCP-1. Monocytes were isolated as indicated in Materials and methods section. Psoriatic individuals’ monocytes were treated with CBD (10μM) or with vehicle alone (DMSO 0,1%) for 18 hours. **(A)** The bar graph shows the flow cytometry histogram profile of CD16, CD68, CD69, CCR7, CXCR3, CD62L, CX3CR1, CCR5 and CCR2 expression at the cell surface of CD14+ monocytes. Pairwise comparisons statistically significant are indicated. **(B)** The histogram shows the percentage of CD14+ monocytes migrating in response to chemotactic stimuli (monocytes’ migration was observed in free medium with addition of MCP-1 0,05 μg/mL or free medium +10% FBS). (ANOVA; **p<0,01; ***p< 0,001).

Next, we wondered if the CBD effects outlined above was limited to NK cell compartment or included other cell components of the innate response arm, e.g. monocytes, usually recruited to the inflammation sites. Firstly, monocytes were isolated from psoriatic individuals and were treated with CBD or with vehicle alone (DMSO 0,1%). Monocytes were characterized by flow cytometry upon gating cells on CD14+ positive cells. Among the explored surface markers, CBD did not impact the monocyte lineage markers (CD16, CD68) nor the cell adhesion molecule CD62L, CX3CR1, CCR2 but it impacted the early activation marker CD69 (CD69: Control: 58% ± 3,5, CBD (10µM) 26% ± 3,2). Among the chemokine and recruitment receptors, CBD downregulates selectively the expression of CCR5 (Control: 62% ± 4,2; CBD (10µM): 8% ± 2,2), with no effect on CCR7, CXCR3, CX3CR1, CCR2. CCR5 plays an important role for the migration of the inflammatory monocytes to tissue (11). Thus, considering the inflammatory status in psoriatic individuals, we moved to evaluate *in vitro* the chemotaxis of psoriatic individuals’ monocytes in response to canonical agents (i.e., MCP) after receiving or not CBD treatment. As expected, the percentage of migrating monocytes in response to 10% FBS or MCP stimuli is significantly reduced by CBD treatment (**Figures 5A**, **B**), confirming what we ascertained through flow cytometry analysis on monocytes.

### 3.3 CBD interferes with maturation of DC, activation of T cell dependent responses and macrophage polarization

**Figure 6:**
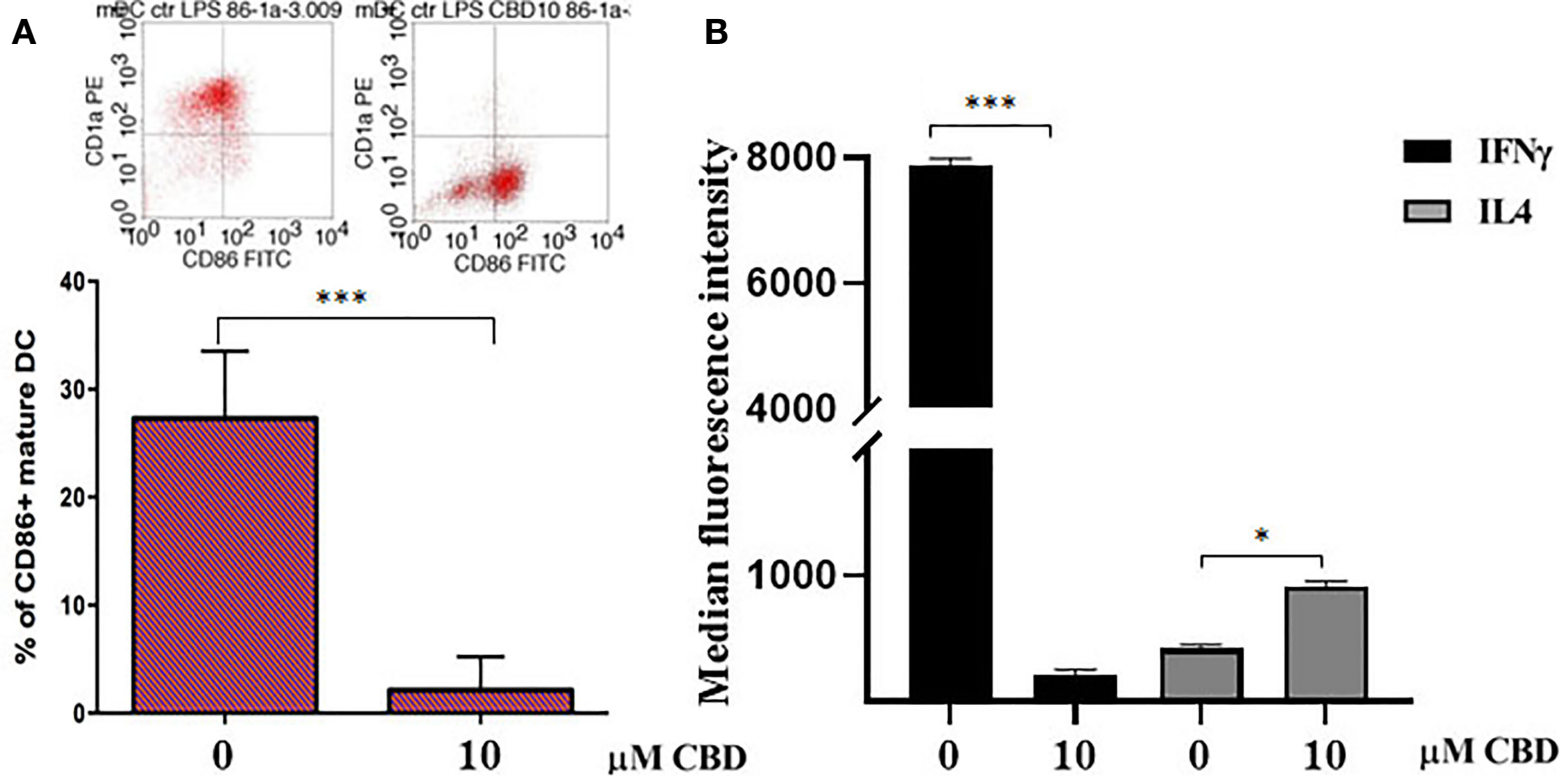
CBD interferes with LPS-induced maturation of DC and activation of T cell dependent responses. **(A)** Monocyte-derived dendritic cells from psoriatic individuals were generated in presence of CBD (10μM) or with vehicle alone (DMSO 0,1%) for 7 days and then exposed to LPS maturation for additional 24h. The bar graph shows the flow cytometry analysis of the maturation surface marker CD86 on CD1a positive monocyte-derived dendritic cells from psoriatic individuals. **(B)** Monocyte-derived dendritic cells from psoriatic individuals were co-cultured with T cells in presence of CBD (10μM) or with vehicle alone (DMSO 0,1%). The histogram highlights the release levels of IFNγ and IL-4 following 7 days-co-culture. (ANOVA; *p<0,05, ***p<0,001).

**Figure 7:**
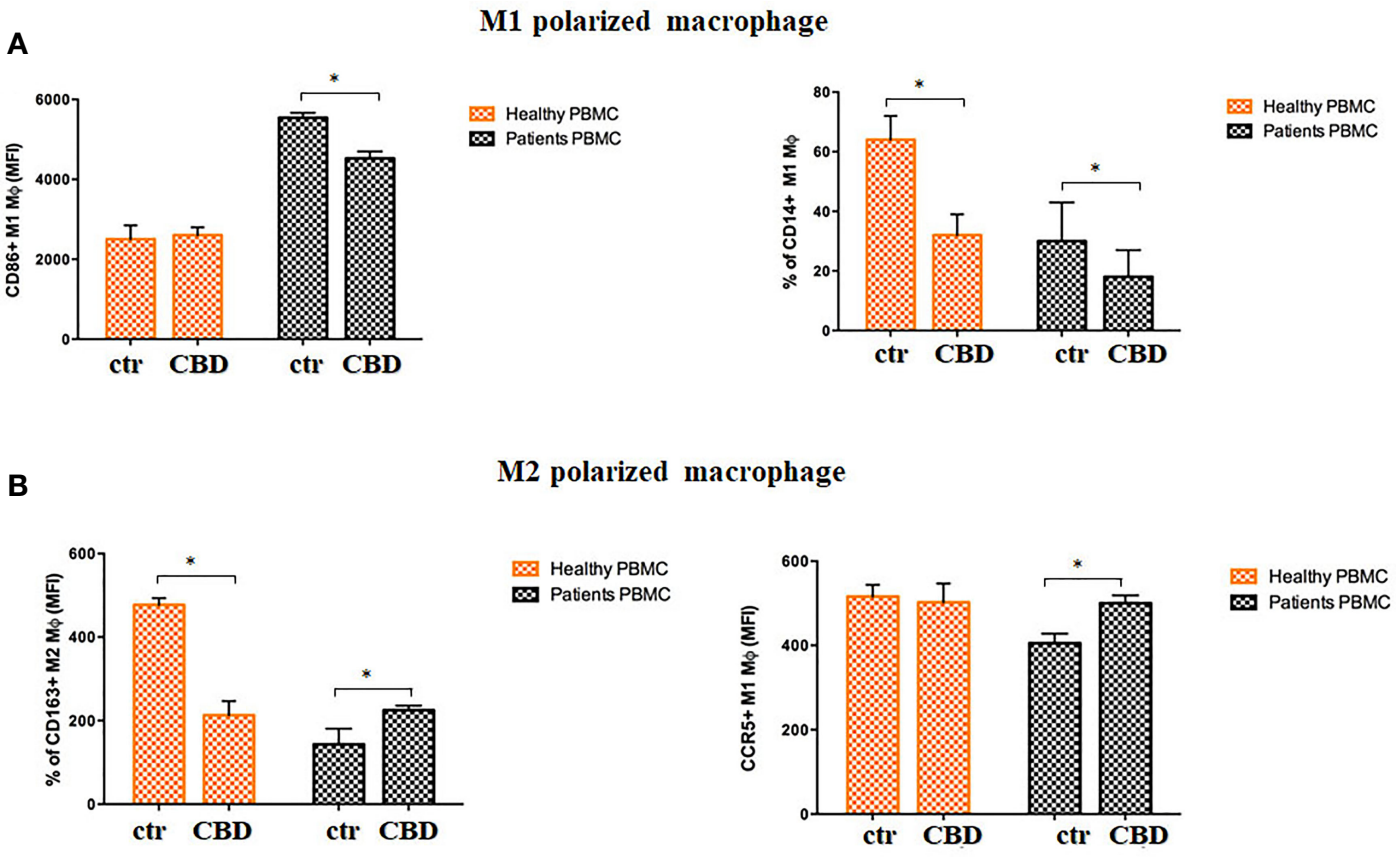
CBD skews monocytes toward M2 pro-resolutive macrophage differentiation. Macrophages were generated from PBMCs of healthy donors and psoriatic individuals upon 7 days *in vitro* culture in presence or absence of CBD (10μM). **(A)** Panel shows the M1 phenotype through the flow cytometry histogram profiles of CD86 (on the left) and CD14 (on the right) protein levels at the cell surface of recovered macrophages of healthy donors and psoriatic individuals. **(B)** Panel shows the M2 phenotype through the flow cytometry histogram profiles of CD163 (on the left) and CCR5 (on the right) protein levels at the cell surface of recovered macrophages of healthy donors and psoriatic individuals. Both bars graph in panel’s reports the mean fluorescence intensity (MFI) values. Data are reported as mean ± SD. (ANOVA; *p<0,05).

Given that CBD interferes with monocytes migration, we wondered if CBD treatment in psoriatic condition could impact on other monocytic function, e.g. their ability to differentiate toward both dendritic cells, and macrophages. Monocyte-derived dendritic cells generated from psoriatic individuals in presence of CBD are unable to undergo to LPS-induced maturation (**Figures 6A**, **B**), as demonstrated by the downregulation of the surface marker CD86 through flow cytometry analysis (CD86: Control: 28,5% ± 5,7; CBD (10µM) 2% ± 3,5). Moreover, in a co-culture T cell and DCs system, CBD was also able to interfere with T cell polarizing capacity of monocyte-derived dendritic cells generated in its presence. This is true considering the reduction of IFNγ (Control: 7900 MFI ± 1,5, CBD (10µM) 210 MFI ± 0,8) coupled with the slight increase of IL-4 levels (Control: 480 MFI ± 0,5, CBD (10µM) 220 MFI ± 0,5), respectively features of Th-1 and Th-2 polarization. Furthermore, upon selected stimuli, monocytes can polarize into pro-inflammatory M1-like (CD14+CD86+) or pro-resolutive M2-like macrophages (CCD163+CCR5+). As shown in **Figures 7A, B**, in M2 committed monocytes differentiated in presence of CBD, cell surface protein expression of CD163, significantly exceed the levels induced by classical M‐CSF stimulation in M2‐like Mφ of psoriatic individuals but not of healthy controls. At the same way, cell surface protein expression of CD14 and CD86 in M1 committed monocytes differentiated in presence of CBD, failed to reach the levels induced by classical GM‐CSF stimulation in M1‐like Mφ of psoriatic individuals. Overall, this evidence suggests a pro-resolutive skewing of the macrophagic response which deserve more attention in the near future.

## 4 Discussion

In psoriasis, there is a complex interaction between innate and adaptive immunity in response to an unidentified trigger. Numerous studies have described the presence of a considerable number of immune cells in psoriatic lesions. Furthermore, keratinocytes, main constituents of the epidermis, plays a critical role in the regulation of immune activities in the skin, responding to antigenic stimuli in a nonspecific manner (12). Therefore, psoriatic lesions probably develop as an interaction between cells and mediators derived by the immune system, and by the dermo-epidermal components (13). In recent years, the possible therapeutic application of cannabinoids, particularly CBD, for the treatment of skin pathologies has attracted a lot of interest, particularly thanks to the demonstrated anti-inflammatory properties. In fact, several evidence show the modulation of immunological signalling pathways by CBD. This compound carries out the anti-inflammatory action through inhibition of JAK/STAT signalling pathway downregulating immune regulatory cytokines, such as TNFα and IFNγ, fundamental to T cell-mediated immunity and adaptive immune responses (14). *In vitro* and *ex vivo* studies show that CBD decreases the activity of the nuclear factor-k B (NF-kB) signalling pathway and of signal transducer and activator of transcription 1 (STAT1) transcription factor, key player in IFN-β-dependent proinflammatory processes (15). Moreover, it has been observed that CBD at 10 μM decreases the production and release of proinflammatory cytokines, including interleukin1, interleukin-6, and interferon (IFN)γ, from LPS-activated microglial cells (16). The anti-inflammatory effect of CBD has been proven in numerous experimental studies and clinical trial, bringing benefits to individuals suffering from dermatitis (13, 17). In this study, we evaluated *in vitro* the CBD immunomodulatory effect in PBMCs of individuals suffering from psoriasis. Using protein arrays, we have observed that the secretion profile of cytokines and growth factors (TARC, MIP, IL-19, IL-1RA, IFNγ, GM-CSF, ENA-78, TNFα) was reduced in CBD-treated PBMCs from affected individuals compared to untreated ones. Indeed, we further evaluated the secretion levels of two cytokines TNFα and IFNγ which, through activation of keratinocytes, drive epidermal hyperproliferation and production of growth factors and chemokines, thus enhancing the inflammatory state in psoriatic lesions (9). We found that CBD, at concentration of 10 μM, reduced the release of IFNγ and TNFα from PBMC of affected individuals activated with phytohemagglutinin (PHA). Such inflammatory cytokines usually regulate the activity of immune cells such as T cells and monocytes, which are known to be involved in the pathogenesis of psoriasis. However, we decided to look at Natural Killer cells response in attempt to try to explore novel responders in the disease pathogenesis. Indeed, recent findings have demonstrated that innate immune cells such as innate lymphoid cells, γδT cells and Natural Killer cells are activated in psoriasis contributing to the development of disease. For example, it has been observed in psoriatic lesions, IFNγ and TNFα produced by NK cells are responsible for keratinocyte activation (18). Moreover, the Natural Killer cells exhibited lower levels of cytotoxicity (14, 19). Here, we have showed that the CBD treatment of NK cells isolated from psoriatic individuals increased the early marker of cell activation CD69 (**Figure 3A**). Furthermore, CBD potentiates the cytotoxic activity of NK cells from psoriatic individuals, but not from healthy donors, as can be seen from the increased expression of CD107a degranulating marker (15, 20). From a functional point of view, while it did not affect the percentage of activated NKG2D positive NK cells of psoriatic individuals (**Figure 3C**), CBD significantly downregulated the expression of the inhibitory receptor KIR3DL1, both in healthy and psoriatic NK cells stimulated by IL-2 (**Figure 3D**). KIR proteins are believed to play an important role in regulating the NK immune response and, particularly KIR3DL1 has been found associated to psoriasis inflammation (16, 21). The reduction of KIR3DL1 in NK cells of psoriatic individuals suggests an increase of the NK immune cell response. To support the hypothesis that the CBD treatment potentiates the NK cells activity, the upregulation of chemokine receptors on NK cells from psoriatic individuals has been found; in detail CXCR3 and CCR5 receptors known to be involved in recruitment and NK cell homing (**Figures 4A, B**). Afterwards, we have observed that monocytes isolated from PBMC of psoriatic individuals treated with CBD lost their functional ability to migrate in response to chemotactic stimuli (i.e. MCP or 10% FBS) compared to cells treated with vehicle alone (DMSO 0,1%) (**Figure 5B**). Indeed, CBD downregulated selectively the expression of CCR5, which plays an important role in the migration of the inflammatory monocytes to tissue (11). More, CBD treatment of monocyte-derived dendritic cells generated from psoriatic individuals did not undergo the LPS-induced maturation, as can be seen from the reduction of the CD86 surface marker. These results are very interesting because recent research have demonstrated that monocyte-derived dendritic cells contribute to the perpetuation of psoriasis, through production of inflammatory cytokines that elevate the activation of skin T cells (22, 23). Subsequent co-culture assays between dendritic cells and autologous T lymphocytes have shown that CBD not only interferes with the full functional maturation of dendritic cells, in response to pathogenic stimuli (Lipopolysaccharide), but modulates their ability to initiate the T lymphocyte response. The DCs generated in the presence of CBD are in fact able to selectively polarize the response of the T helper lymphocytes in the Th2 direction, allowing a better control of the inflammatory process. Furthermore, upon selected stimuli, monocytes can polarize into pro-inflammatory M1-like (CD14+CD86+) or pro-resolutive M2-like macrophages (CCD163+CCR5+). CBD treatment (**Figure 7**) increased the yield of the anti-inflammatory M2 macrophages in psoriatic individuals but not in healthy control, and contemporary reduced the yield of pro-inflammatory M1 macrophages, suggesting a pro-resolutive skewing of the macrophagic response. From studies it emerges that M1 macrophages contribute to the development of psoriasis, through production of TNFα (24). Finally, the characterization by flow cytometry of the phenotype of macrophages generated in the presence of CBD has also showed that this cell population is characterized by a higher expression of receptors/”scavenger” molecules (CD163+) (**Figure 7**), suggesting that macrophage targeting could be useful for removing the products of epidermal degeneration, with improvement of the psoriatic loop.

These interesting data describe the effect of cannabidiol on the PBMC of individuals suffering from psoriasis, however the mechanism through which it carries out its action remains elusive. Several evidence reveal that CBD has beneficial effects for a variety of acute and chronic disorders such as autoimmune diseases, inflammation, tissue repair processes or increased oxidative damage. In Multiple Sclerosis (MS), CBD in preclinical models of MS resulted in a reduction of proinflammatory cytokines such as IL-17A, IFN-γ, TNF-α, IL-6 and IL-1b and an increase in anti-inflammatory cytokines such as IL-4, IL-10 and TGF-β. Data available in the literature do not report any involvement of receptors such as CB1, CB2, 5-HT1A, TRPV1 or PPARγ in the CBD-dependent reduction of IL-17 secretion or of CB1, CB2 or GPR55 in the inhibition of IL-6 secretion. Currently, the molecular targets involved in the effects of CBD in EAE and possibly MS are not yet determined (25). In allergic contact dermatitis, CBD reduces IL-6, TNF-α and monocyte chemotactic protein-2 (MCP-2) in in poly-(I:C)–stimulated HaCaT cells, an *in vitro* model of this disorder. This effect of CBD is mediated by CB2 and TRPV1 receptors, as the protective effects of CBD are blocked by antagonists of these receptors (26). CBD can exert its anti-inflammatory effects through TRPV1, 5-HT1A, PPARγ receptors. Ultimately, CBD can exert its action through different cellular mechanisms, but precise mechanisms of action remain unclear. This study represents a characterization of the immunomodulatory effects of CBD on PBMC of individuals suffering from psoriasis. We are currently studying the mechanism of action of CBD and which types of receptors may be involved.

In conclusion, although the sample size is small, the data obtained are significant and represent preliminary and suggestive evidence on the modulatory effect of CBD on various immune cells active in psoriatic lesions. In the future, we will carry out other experiments to evaluate the topical application of CBD both in mice and on affected subjects. As already demonstrated for other skin diseases, such as dermatitis, CBD could represent a valid therapeutic option in the treatment of this disease.

## Data availability statement

The raw data supporting the conclusions of this article will be made available by the authors, without undue reservation.

## Ethics statement

The study was approved by the local ethical committee (prot num. 0037908 year 2021) and conducted in accordance with the ethical principles deriving from the Declaration of Helsinki. Written informed consent has been obtained from the patients to publish this paper.

## Author contributions

CP: Conceptualization, Data curation, Writing – original draft, Formal analysis, Supervision, Writing – review & editing. EC: Conceptualization, Data curation, Investigation, Writing – original draft, Formal analysis, Supervision, Writing – review & editing. LC: Data curation, Investigation, Writing – review & editing. VL: Data curation, Investigation, Writing – review & editing. AR: Investigation, Writing – review & editing. MG: Writing – review & editing. SL: Data curation, Investigation, Writing – review & editing. CL: Conceptualization, Writing – original draft, Writing – review & editing, Formal analysis, Supervision. MB: Writing – review & editing, Funding acquisition, Supervision, Validation.

## Supplementary material

Supplementary Figure 1 The graph represents the average of two experiments conducted on a human keratinocytes cell line (HaCat cells). Cells were treated with increasing concentrations of CBD (1.5µM - 40µM) for 24, 48, 72 h of treatment. We found no changes in the proliferation rate (IC50 20µM) for concentration lower than 15 µM.

## References

[1] Parisi R Symmons DP Griffiths CE Ashcroft DM Identification and Management of Psoriasis and Associated ComorbidiTy (IMPACT) project team. Global epidemiology of psoriasis: a systematic review of incidence and prevalence. J Invest Dermatol. (2013) 133:377–85. doi: 10.1038/jid.2012.339 23014338

[2] Ogawa E Sato Y Minagawa A Okuyama R. Pathogenesis of psoriasis and development of treatment. J Dermatol. (2018) 45:264–72. doi: 10.1111/1346-8138.14139 29226422

[3] Alwan W Nestle FO. Pathogenesis and treatment of psoriasis: Exploiting pathophysiological pathways for precision medicine. Clin Exp Rheumatol. (2015) 33:S2–6. 26472336

[4] Baswan SM Klosner AE Glynn K Rajgopal A Malik K Yim S. Therapeutic potential of cannabidiol (CBD) for skin health and disorders. Clin Cosmet Investig Dermatol. (2020) 13:927–42. doi: 10.2147/CCID.S286411 PMC7736837 33335413

[5] Tóth KF Ádám D Bíró T Oláh A. Cannabinoid signaling in the skin: therapeutic potential of the "C(ut)annabinoid" System. Molecules. (2019) 24:918. doi: 10.3390/molecules24050918 30845666 PMC6429381

[6] Jarocka-Karpowicz I Biernacki M Wroński A Gęgotek A Skrzydlewska E. Cannabidiol effects on phospholipid metabolism in keratinocytes from patients with psoriasis vulgaris. Biomolecules. (2020) 10:367. doi: 10.3390/biom10030367 32121131 PMC7175188

[7] Scheau C Badarau IA Mihai LG Scheau AE Costache DO Constantin C. Cannabinoids in the pathophysiology of skin inflammation. Molecules. (2020) 25:652. doi: 10.3390/molecules25030652 32033005 PMC7037408

[8] Martinez Naya N Kelly J Corna G Golino M Abbate A Toldo S. Molecular and cellular mechanisms of action of cannabidiol. Molecules. (2023) 28:5980. doi: 10.3390/molecules28165980 37630232 PMC10458707

[9] Griffiths CEM Armstrong AW Gudjonsson JE Barker JNWN. Psoriasis. Lancet. (2021) 397:1301–15. doi: 10.1016/S0140-6736(20)32549-6 33812489

[10] Blevins LK Bach AP Crawford RB Zhou J Henriquez JE Rizzo MD. Evaluation of the anti-inflammatory effects of selected cannabinoids and terpenes from Cannabis Sativa employing human primary leukocytes. Food Chem Toxicol. (2022) 170:113458. doi: 10.1016/j.fct.2022.113458 36228902

[11] Castanheira FVES de Lima KA Cebinelli GCM Sônego F Kanashiro A Colon DF. CCR5-positive inflammatory monocytes are crucial for control of sepsis. Shock. (2019) 52:e100–6. doi: 10.1097/SHK.0000000000001301 30724784

[12] Perera GK Di Meglio P Nestle FO. Psoriasis. Annu Rev Pathol. (2012) 7:385–422. doi: 10.1146/annurev-pathol-011811-132448 22054142

[13] Nestle FO Kaplan DH Barker J. Psoriasis. N Engl J Med. (2009) 361:496–509. doi: 10.1056/NEJMra0804595 19641206

[14] Peyravian N Deo S Daunert S Jimenez JJ. Cannabidiol as a novel therapeutic for immune modulation. Immunotargets Ther. (2020) 9:131–40. doi: 10.2147/ITT.S263690 PMC7445536 32903924

[15] Pisanti S Malfitano AM Ciaglia E Lamberti A Ranieri R Cuomo G. Cannabidiol: State of the art and new challenges for therapeutic applications. Pharmacol Ther. (2017) 175:133–50. doi: 10.1016/j.pharmthera.2017.02.041 28232276

[16] Kozela E Pietr M Juknat A Rimmerman N Levy R Vogel Z. Cannabinoids Delta(9)- tetrahydrocannabinol and cannabidiol differentially inhibit the lipopolysaccharide-activated NF-kappaB and interferon-beta/STAT proinflammatory pathways in BV-2 micrglial cells. J Biol Chem. (2010) 285:1616–26. doi: 10.1074/jbc.M109.069294 PMC2804319 19910459

[17] Yoo EH Lee JH. Cannabinoids and their receptors in skin diseases. Int J Mol Sci. (2023) 24:16523. doi: 10.3390/ijms242216523 38003712 PMC10672037

[18] Ottaviani C Nasorri F Bedini C de Pità O Girolomoni G Cavani A. CD56brightCD16(-) NK cells accumulate in psoriatic skin in response to CXCL10 and CCL5 and exacerbate skin inflammation. Eur J Immunol. (2006) 36:118–28. doi: 10.1002/eji.200535243 16323244

[19] Dunphy SE Sweeney CM Kelly G Tobin AM Kirby B Gardiner CM. Natural killer cells from psoriasis vulgaris patients have reduced levels of cytotoxicity associated degranulation and cytokine production. Clin Immunol. (2017) 177:43–9. doi: 10.1016/j.clim.2015.10.004 26477484

[20] Aktas E Kucuksezer UC Bilgic S Erten G Deniz G. Relationship between CD107a expression and cytotoxic activity. Cell Immunol. (2009) 254:149–54. doi: 10.1016/j.cellimm.2008.08.007 18835598

[21] Ahn RS Moslehi H Martin MP Abad-Santos M Bowcock AM Carrington M. Inhibitory KIR3DL1 alleles are associated with psoriasis. Br J Dermatol. (2016) 174:449–51. doi: 10.1111/bjd.14081 PMC4752910 26286807

[22] Singh TP Zhang HH Borek I Wolf P Hedrick MN Singh SP. Monocyte-derived inflammatory Langerhans cells and dermal dendritic cells mediate psoriasis-like inflammation. Nat Commun. (2016) 7:13581. doi: 10.1038/ncomms13581 27982014 PMC5171657

[23] Pénzes Z Alimohammadi S Horváth D Oláh A Tóth BI Bácsi A. The dual role of cannabidiol on monocyte-derived dendritic cell differentiation and maturation. Front Immunol. (2023) 14:1240800. doi: 10.3389/fimmu.2023.1240800 37680639 PMC10482398

[24] Kamata M Tada Y. Dendritic cells and macrophages in the pathogenesis of psoriasis. Front Immunol. (2022) 13:941071. doi: 10.3389/fimmu.2022.941071 35837394 PMC9274091

[25] Furgiuele A Cosentino M Ferrari M Marino F. Immunomodulatory potential of cannabidiol in multiple sclerosis: a systematic review. J Neuroimmune Pharmacol. (2021) 16:251–69. doi: 10.1007/s11481-021-09982-7 PMC7829325 33492630

[26] Etemad L Karimi G Alavi MS Roohbakhsh A. Pharmacological effects of cannabidiol by transient receptor potential channels. Life Sci. (2022) 300:120582. doi: 10.1016/j.lfs.2022.120582 35483477

