# Supplementary figures and images for: Cannabidiol exerts multitarget immunomodulatory effects on PBMCs from individuals with psoriasis vulgaris
Source: Front Immunol. 2024 Mar 27;15:1373435. doi: 10.3389/fimmu.2024.1373435 (PMC11004238; doi:10.3389/fimmu.2024.1373435)

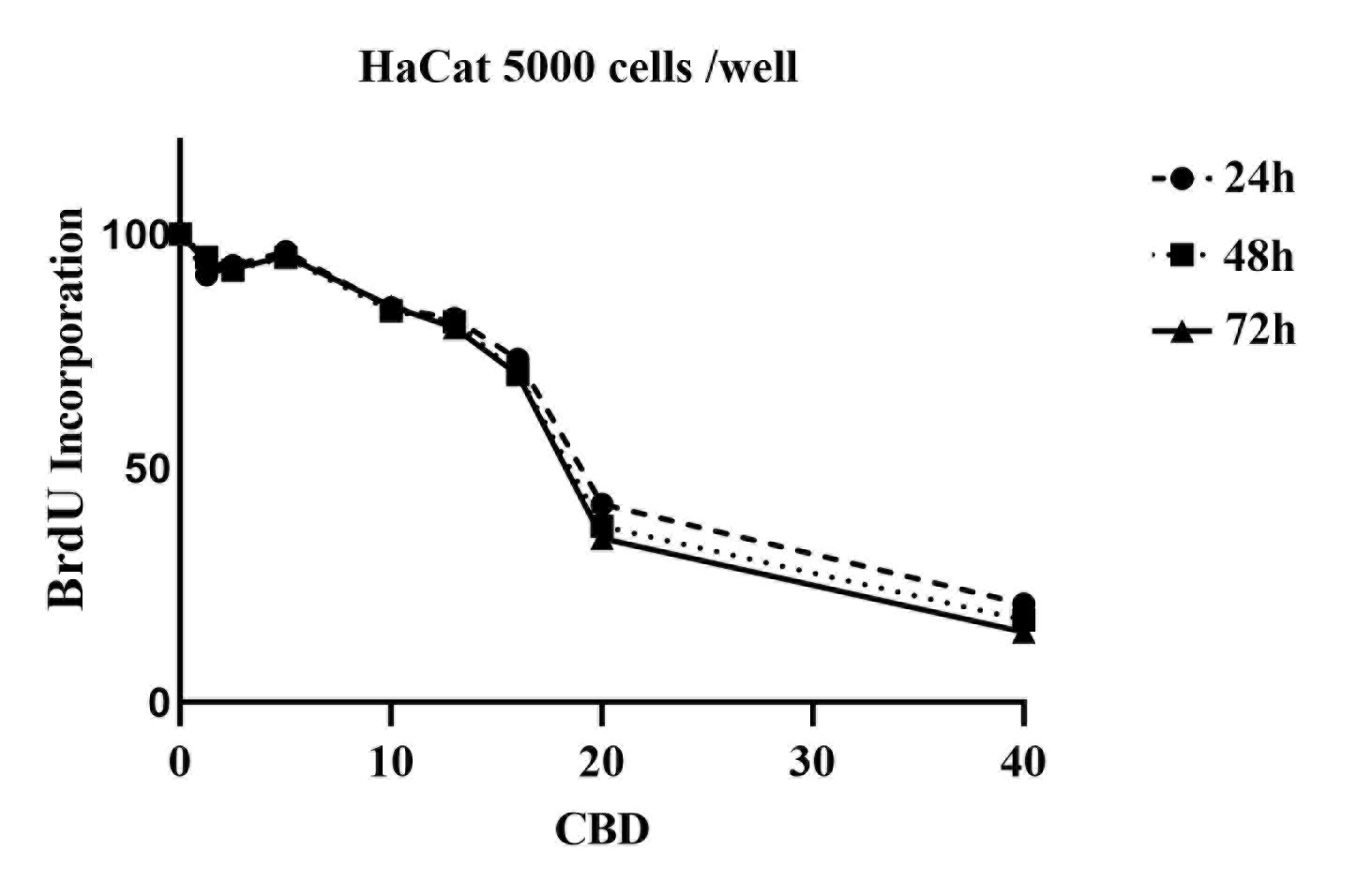

Supplement: Supplementary Figure 1 — The graph represents the average of two experiments conducted on a human keratinocytes cell line (HaCat cells). Cells were treated with increasing concentrations of CBD (1.5µM - 40µM) for 24, 48, 72 h of treatment. We found no changes in the proliferation rate (IC50 20µM) for concentration lower than 15 µM. [file Image_1.tif]
